# Who Uses Physician-Rating Websites? Differences in Sociodemographic Variables, Psychographic Variables, and Health Status of Users and Nonusers of Physician-Rating Websites

**DOI:** 10.2196/jmir.3145

**Published:** 2014-03-31

**Authors:** Ralf Terlutter, Sonja Bidmon, Johanna Röttl

**Affiliations:** ^1^Department of Marketing and International ManagementAlpen-Adria Universitaet KlagenfurtKlagenfurt am WoertherseeAustria

**Keywords:** physician-rating websites, sociodemographic variables, psychographic variables, digital literacy

## Abstract

**Background:**

The number of physician-rating websites (PRWs) is rising rapidly, but usage is still poor. So far, there has been little discussion about what kind of variables influence usage of PRWs.

**Objective:**

We focused on sociodemographic variables, psychographic variables, and health status of PRW users and nonusers.

**Methods:**

An online survey of 1006 randomly selected German patients was conducted in September 2012. We analyzed the patients’ knowledge and use of online PRWs. We also analyzed the impact of sociodemographic variables (gender, age, and education), psychographic variables (eg, feelings toward the Internet, digital literacy), and health status on use or nonuse as well as the judgment of and behavior intentions toward PRWs. The survey instrument was based on existing literature and was guided by several research questions.

**Results:**

A total of 29.3% (289/986) of the sample knew of a PRW and 26.1% (257/986) had already used a PRW. Younger people were more prone than older ones to use PRWs (*t*_967_=2.27, *P*=.02). Women used them more than men (χ^2^_1_=9.4, *P*=.002), the more highly educated more than less educated people (χ^2^_4_=19.7, *P*=.001), and people with chronic diseases more than people without (χ^2^_1_=5.6, *P*=.02). No differences were found between users and nonusers in their daily private Internet use and in their use of the Internet for health-related information. Users had more positive feelings about the Internet and other Web-based applications in general (*t*_489_=3.07, *P*=.002) than nonusers, and they had higher digital literacy (*t*_520_=4.20, *P*<.001). Users ascribed higher usefulness to PRWs than nonusers (*t*_612_=11.61, *P*<.001) and users trusted information on PRWs to a greater degree than nonusers (*t*_559_=11.48, *P*<.001). Users were also more likely to rate a physician on a PRW in the future (*t*_*367*_=7.63, *P*<.001) and to use a PRW in the future (*t*_619_=15.01, *P*<.001). The results of 2 binary logistic regression analyses demonstrated that sociodemographic variables (gender, age, education) and health status alone did not predict whether persons were prone to use PRWs or not. Adding psychographic variables and information-seeking behavior variables to the binary logistic regression analyses led to a satisfying fit of the model and revealed that higher education, poorer health status, higher digital literacy (at the 10% level of significance), lower importance of family and pharmacist for health-related information, higher trust in information on PRWs, and higher appraisal of usefulness of PRWs served as significant predictors for usage of PRWs.

**Conclusions:**

Sociodemographic variables alone do not sufficiently predict use or nonuse of PRWs; specific psychographic variables and health status need to be taken into account. The results can help designers of PRWs to better tailor their product to specific target groups, which may increase use of PRWs in the future.

## Introduction

### Development of Physician-Rating Websites

In recent years, there has been increasing interest in online rating websites, which have become part of life for many of us [1]. Web 2.0 has supported the enormous growth of online rating websites [2] and online reviews tend to shift the balance of authority in the doctor-patient relationship [3]. Physician-rating websites (PRWs) are structured in a similar way to other existing rating sites (eg, travel-, hotel-, or restaurant-rating sites). Patients can rate and discuss the quality of their physicians online [4]. Rating sites for professional services or restaurants and hotels are already widespread and well known, but this is a relatively new Web-based tool in the area of medicine. For instance, PRWs provide information about a physician’s address, business hours, and certifications. However, the most important goal of a PRW is to rate and discuss a physician’s quality [5].

Examples of PRWs are RateMDs [6], Vitals [7], ZocDoc [8], and jameda [3,9,10-13]. Some sites are free (eg, RateMDs), whereas other sites provide some free information with more detailed information provided for a fee (eg, Healthgrades [14]) [15,16]. By now, 1 in 6 physicians have already been rated, with 90% of all ratings being positive [10].

Experts’ opinions differ on whether the standard of care in the future will improve or not [3]. Since 2001, the Bertelsmann Health Care Monitor, a periodically conducted survey financed by the German Bertelsmann foundation, has been collecting data from a representative sample of the German population aged between 18 and 79 years on diverse aspects of health care. According to the 2009 Bertelsmann Health Care Monitor [17], there has been an emerging trend to use the Internet for searching for a general practitioner (GP), for example. In 2007, 5% of 1464 representative selected respondents in the German population had used the Internet to search for a GP, and that percentage had risen to 9% in 2009 [18]. The percentage had increased even higher for searching for a medical specialist (8% in 2007 to 13% in 2009). According to a recent study conducted by Gesellschaft für Konsumforschung (GfK) HealthCare, a leading survey research company in Nuremberg, Germany, almost 23% of German Internet users trust PRWs when looking for a physician online [19]. Emmert et al [20] conducted a study in Germany in January 2013 that showed that 32% of an online panel were aware of PRWs, 25% of respondents had used a PRW in the past when searching for a physician, and 11% had rated a physician on a PRW at least once. Another important result of this study was that 65% of those who received information from a PRW consulted a physician based on the provided rating, whereas 17% had not consulted a particular physician because of the results found on a PRW [20]. In 2012, Galizzi et al [21] investigated the awareness and use of PRWs in a borough of London, United Kingdom. They calculated that 15% of the respondents (a convenience sample of the general population) were aware of the existence of PRWs [19]. In the United States, usage of PRWs is also still poor [4].

The PRWs seem to offer many advantages for patients. They may provide them with important information, help patients to find physicians with a particular style, help them in their decision-making process, and make them better prepared for their future visits to doctors. PRWs may even improve standards of care and promote trustful doctor-patient relationships [22]. Patients are more likely to rely on PRWs if the information they find is specific to their needs [23]. Some disadvantages are also obvious: physicians fear that PRWs encourage negative reviews. However, an analysis of health care providers’ online ratings showed that online ratings were largely positive [24]. Additionally, too few ratings on a site raise concerns—especially from physicians and health organizations—over the representativeness of judgments and scientific validity [3,4]. In general, physicians tend to be skeptical about the quality of health information on the Internet, which is in-line with existing empirical studies assessing the quality of health information on the Internet [25]. If patients are looking for structural information (eg, services offered, opening hours, office location) rather than process or outcome measures, PRWs are successful [5]. PRWs are able to deliver information, but PRWs also cause misinformation and present risks for the rated person in terms of outcome measures [11].

One important question raised is whether patients are able to evaluate the medical expertise and capabilities of a doctor [26]. It should also be considered that, for instance, an analysis of German PRWs demonstrates that the average number of ratings per physician available on PRWs is still poor. For the ratings website jameda [9], which has the largest number of total ratings, an average of 4 evaluations per physician could be found [5]. A recent study in Great Britain found an average of 2 ratings per practice over a 15-month period for a PRW funded by the government [27]. Gao et al [28] reported that only 1 in 6 physicians included in their comprehensive analysis of national ratings in the United States between 2005 and 2010 had been rated on a PRW. In Germany in 2011, a new PRW was created called “Weisse Liste.” More than 30 million Germans insured by AOK, BARMER GEK, and other statutory health insurance funds were invited to rate their physicians [29]. In contrast to other PRWs, this Internet portal is noncommercial and free of ads, insured individuals are invited to participate, and a secure registration and authentication procedure prevents misuse of data [29]. Therefore, this site is one of the first to meet most of the demands of quality criteria for PRWs [30,31]. According to Emmert et al [5], further relevance of PRWs can be assumed for other reasons, such as an increasing number of websites [18] and the rapid spread of Web 2.0 services [32].

### Health Information Search

Health is one of the most searched topics online [17]. In 2011, 4 out of 5 adult Internet users in the United States searched for health information on the Internet [17,18]. Some demographic groups are more likely to look online for health information than others. Adults between the ages of 18-49 years, for example, are more likely to seek health information online than older people. Less than half of the adults in the United States aged 65 years and older use the Internet to look for health information [33]. With regard to gender, women are more likely to look online for health-related information than men [33-36]. Almost 90% of people with a college degree use the Internet to gather information online, compared to 62% of those with a high school education [33,35]. People with a chronic disease or disability are more interested in online health information than people without a chronic disease or disability [37]. Approximately one-quarter of all Internet users in the United States who use the Internet for health- and medical-related information look at PRWs [23,37], and 30% of adult American patients compare physicians online before making a selection. Regarding data obtained from the RateMDs website, 16% of national physicians were rated online in the United States [10,13]. Almost 18% of patients with a chronic disease have looked for physician rankings or reviews online and 6% of patients with a chronic disease have already posted a review [37]. A recent US study showed that online reviewers of social care review sites tend to be younger, healthier, and more affluent than health care users overall [3].

However, little is known about variables that influence usage of PRWs. The question arises how the previously mentioned variables analyzed in the health information literature along with additional psychographic variables can be related to the use of PRWs. The objectives of this research are to:

1. Analyze patients’ knowledge and use of PRWs,
2. Describe users and nonusers in terms of sociodemographic variables, psychographic variables, and health status, and
3. Assert whether these variables can also serve as predictors of usage and nonusage of PRWs.

Results of this paper will be useful for the improvement of PRWs as they help to tailor PRWs more closely to the needs of users. In addition to sociodemographic variables (gender, age, education), this paper also takes psychographic variables (eg, feelings toward the Internet, digital literacy) and health status into account.

## Methods

### Participant Recruitment

An online survey of 1006 German patients was conducted in September 2012. The term “patients” in this paper refers to individuals that have visited a physician at least once in the previous 3 months. The sample was drawn from an e-panel maintained by GfK HealthCare, a leading survey research company in Nuremberg, Germany. It was based on a randomly generated set of users who had visited a GP at least once during the 3 months before the beginning of the survey. Originally, 1561 individuals were contacted; 555 persons could not participate because they did not fulfill this criterion. The recruitment rate was 64.45% (1006/1561) [38]. In all, 20 participants were excluded from the analysis because of an extremely short answer time and/or inconsistent answer patterns (eg, flatliners, contradictions) resulting in 986 usable respondents. Small monetary incentives were offered for survey completion.

### Questionnaire

The survey was designed by the researchers based on the existing literature and was guided by the research questions. All items apart from categorical variables were measured with 7-point rating scales (see Multimedia Appendix 1). All items had a “no answer” category as an alternative. Existing scales from the literature were used where applicable. Data were analyzed using SPSS version 20 (IBM Corp, Armonk, NY, USA).

### Measurement of Physician-Rating Website Items

#### Knowledge of Physician-Rating Websites

To assess whether respondents knew about PRWs, they were explicitly asked if they knew any online physician-rating platforms with the following question: “Do you know websites on which patients have the opportunity to rate their physicians?” (1=yes; 2=no). To ensure that the respondents understood the term “PRW,” an example of a rating platform was given as an introductory phrase before asking the actual question.

#### Usage of Physician-Rating Websites

Respondents were asked if they had gathered information at least once on a PRW with the question: “Have you ever gathered information on a physician from a PRW?” (1=yes; 2=no). Additionally, they were asked whether they had rated a physician on an online rating platform like this before (1=yes; 2=no). All respondents with a negative response to this question were asked additionally whether they could imagine rating on an online physician-rating platform (1=cannot imagine this at all; 7=can imagine this very well) and how probable it is that they would use online physician-rating platforms in the future (1=not at all probable; 7=very probable).

#### Usefulness of Physician-Rating Websites

The usefulness of online physician-rating platforms was judged in comparison to other recommendation sources, such as physicians, family, and friends with the following question: “How useful are PRWs in comparison to other recommendation sources (eg, other physicians, family, friends) from your point of view?” (1=not at all useful; 7=very useful).

#### Trust in Physician-Rating Websites

To examine respondents’ trust in PRWs, we asked whether they trusted the information on online physician-rating platforms with the following question: “How much do you trust the information on PRWs?” (1=no trust at all; 7=very high trust).

### Measurement of Sociodemographic and Psychographic Variables

#### Sociodemographic Variables

Age was measured through the inquiry about the participant’s year of birth and education through the highest completed level of education. The respondents were asked whether they had a chronic disease or not (1=yes; 2=no; 3=no answer).

#### Feelings About the Internet and Other Web-Based Applications

Feelings about the Internet and other Web-based applications in general were measured with the following question: “What kind of feelings do you have toward the Internet and other Web-based applications (eg, apps on smartphones or tablets) in general?” (1=very negative; 7=very positive; 8=no answer).

#### Digital Literacy

Digital literacy is the ability to effectively and critically use a range of digital technologies. Literate individuals are able to make responsible choices and to access information and ideas in the digital world and to share information with others. High levels of digital literacy are seen as an important prerequisite in today’s digital world [39]. Digital literacy was measured with an item based on Norman and Skinner [40]. The respondents were asked the following: “How would you rate your own Internet skills?” (1=not literate at all; 7=very literate).

#### Daily Internet Use

Concerning daily Internet use, respondents were asked how many hours on average on a daily, weekly, or monthly basis they spend on the Internet for private purposes (total private use) and searching for health-related information (total private use for health-related information). Respondents should choose 1 of 3 options (daily, weekly, monthly). We then calculated the total private use of Internet and the total private use of Internet for health-related information for each respondent on a daily basis.

#### Importance of Different Sources for Health-Related Information

Respondents were asked to state the importance of the following single sources for health-related information search on a 7-point scale (1=not important at all; 7=very important; 8=no answer): family, friends, physician, pharmacist, insurance agent, Internet, books/journals, and other sources.

## Results

### Definition of Users and Nonusers

To compare the sociodemographic variables of users and nonusers of PRWs, the respondents were split into 2 groups: those who did have experience with PRWs (users) and those who did not (nonusers). With regard to knowledge about PRWs, 29.3% (289/986) of respondents answered that they knew of PRWs, 68.1% (671/986) of respondents did not know of PRWs, and 2.6% (26/986) of respondents chose the alternative response (no answer). To identify usage of PRWs, respondents were asked whether they had used PRWs for an information search at least once. In all, 26.1% (257/986) of respondents said yes (named users of PRWs), 72.2% (712/986) of respondents said no (named nonusers of PRWs), and 1.7% (17/986) of respondents chose the alternative response (no answer) and were excluded from the analyses.

### Differences Between Users and Nonusers

Table 1 presents the results from the descriptive analysis of chi-square test for users and nonusers of PRWs regarding gender, education, and health status and of *t* test for age.

As shown in Table 1, there are significant differences between men and women in their experience with PRWs. More women than men had used them in the past (χ^2^_1_=9.4, *P*=.002). More respondents with a higher education entrance qualification (eg, people with a high school diploma or people who have graduated from university) had experience with PRWs (χ^2^_4_=19.7, *P*=.001) and younger respondents had experience with gathering information through PRWs (*t*_967_=2.27, *P*=.02). Finally, significantly more participants with chronic disease(s) had used information from a PRW than those without chronic disease(s) (χ^2^_1_=5.6, *P*=.02).

**Table 1:** Differences between users and nonusers of physician-rating websites (PRWs) in reference to sociodemographic variables and health status.

Variables		Users n=257	Nonusers n=712	Total N=969	χ^2^ (*df*)	*t* (*df*)	*P*
Age (years), mean (SD)		42.28 (12.92)	44.42 (12.99)	43.85 (13.00)		2.27 (967)	.02
**Gender, n (%)**					9.4 (1)		.002
	Male	118 (22.5)	406 (77.5)	524 (100)			
	Female	139 (31.2)	306 (68.8)	445 (100)			
**Education,** ^a^ **n (%)**					19.7 (4)		.001
	Without school qualification	2 (50.0)	2 (50.0)	4 (100)			
	Secondary general school	2 (16.7)	10 (83.3)	12 (100)			
	Polytechnic secondary school	14 (11.7)	106 (88.3)	120 (100)			
	Intermediate secondary school	71 (25.4)	208 (74.6)	279 (100)			
	Matura examination or higher	166 (30.4)	380 (69.6)	546 (100)			
**Health status,** ^b^ **n (%)**					5.6 (1)		.02
	No chronic disease	122 (23.6)	396 (76.4)	518 (100)			
	Chronic disease	131 (30.4)	300 (69.6)	431 (100)			

^a^Users: n=255; nonusers: n=706; total: n=961.

^b^Users: n=253; nonusers: n=696; total: n=949.

### Differences Between Users and Nonusers: Psychographic Variables

Table 2 provides the corresponding results of unrelated *t* tests for the psychographic variables and information-seeking behavior variables. As can be seen from the data, there was a significant difference between the 2 groups in their feelings toward the Internet and other Web-based applications in general (*t*_489_=3.07, *P*=.002) and their digital literacy (*t*_520_=4.20, *P*<.001). Users had more positive feelings about the Internet and other Web-based applications than nonusers and had higher competence in digital literacy. When the participants were asked to evaluate the importance of different sources for health-related information, users of PRWs rated the Internet higher than nonusers of PRWs and they rated books or journals and other sources (not specified in the questionnaire) lower than nonusers (see Table 2 for details). The 2 groups did not differ in their daily Internet use measured by the daily hours spent online for private use and, in particular, for using the Internet for health-related information.

Further unrelated *t* tests were used to analyze variables concerning PRWs: usefulness and trust in PRWs and future behavior intentions of the 2 groups. In terms of judging the usefulness of PRWs compared with other recommendation sources, the 2 groups differed as expected (*t*_612_=11.61, *P*<.001) with users ascribing higher usefulness. It can be seen from the data in Table 3 that users trusted information on PRW sites to a greater extent than nonusers (*t*_559_=11.48, *P*<.001) and were more prone to rate a physician online in the future (*t*_367_=7.63, *P*<.001) as well as use PRWs in the future (*t*_619_=15.01, *P*<.001).

**Table 2:** Differences of users and nonusers of physician-rating websites (PRWs) in reference to psychographic variables and information-seeking behavior variables.

Variables		Users (n=257)		Nonusers (n=712)		Total (N=969)		*t* (*df*)	*P*
		n	Mean (SD)	n	Mean (SD)	n	Mean (SD)		
Feelings about the Internet and other Web-based applications in general (1=very negative, 7=very positive)		254	5.96 (1.02)	702	5.72 (1.12)	956	5.78 (1.10)	3.07 (489)	.002
Digital literacy (1=not literate at all, 7=very literate)		257	6.08 (0.95)	712	5.78 (1.10)	969	5.86 (1.06)	4.20 (520)	<.001
**Daily Internet use (hours)**									
	Total private use	257	3.17 (2.04)	712	3.04 (2.34)	969	3.07 (2.27)	0.78 (967)	.43
	Total private use for health-related information	257	0.55 (1.82)	712	0.39 (1.45)	969	0.43 (1.56)	1.47 (967)	.14
**Importance of different sources for health-related information (1=not important at all, 7=very important)**									
	Family	256	4.77 (1.70)	703	4.87 (1.73)	959	4.84 (1.73)	–0.81 (957)	.41
	Friends	252	4.27 (1.70)	703	4.13 (1.75)	955	4.17 (1.74)	1.13 (953)	.26
	Physician	256	6.40 (0.88)	709	6.42 (0.99)	965	6.42 (0.96)	–0.35 (963)	.73
	Pharmacist	248	4.93 (1.56)	699	5.05 (1.58)	947	5.02 (1.57)	–0.98 (945)	.33
	Insurance agent	236	1.82 (1.46)	664	1.73 (1.27)	900	1.75 (1.32)	0.95 (898)	.34
	Internet	256	5.08 (1.19)	707	4.38 (1.50)	963	4.57 (1.46)	7.47 (569)	<.001
	Books/journals	248	4.64 (1.62)	684	4.10 (1.69)	932	4.24 (1.69)	4.32 (930)	<.001
	Other sources	161	3.26 (1.89)	480	2.75 (1.75)	641	2.88 (1.80)	3.16 (639)	.002

**Table 3:** Differences of users and nonusers of physician-rating websites (PRWs) in reference to variables concerning PRWs.

Variables	Users (n=257)		Nonusers (n=712)		Total (N=969)		*t* (df)	*P*
	n	Mean (SD)	n	Mean (SD)	n	Mean (SD)		
Usefulness of PRWs (1=not at all useful, 7=very useful)	254	5.24 (1.45)	692	3.88 (1.98)	946	4.24 (1.95)	11.61 (612)	<.001
Trust of information on PRWs (1=no trust at all, 7=very high trust)	252	4.45 (1.30)	692	3.26 (1.65)	944	3.58 (1.65)	11.48 (559)	<.001
Willingness to rate a physician on a PRW (1=not willing at all, 7=very willing)	193	5.27 (1.74)	678	4.13 (2.11)	871	4.38 (2.09)	7.63 (367)	<.001
Probability of using a PRW in the future (1=not probable at all, 7=very probable)	255	5.46 (1.44)	701	3.69 (1.99)	956	4.17 (2.02)	15.01 (619)	<.001

### Causal Relationship Between the Variables: Binary Logistic Regression

Table 4 shows the results of 2 binary logistic regressions. In a first step, a binary logistic regression (n=942) with the user and nonuser distinction (usage=1; nonusage=0) as criterion and the variables gender, age, education, and health status was carried out. All the remaining variables were entered into the regression in their original form, which means that gender and health status were scaled nominally, education ordinally, and age was scaled metrically. However, our model did not reveal significant fit. Nagelkerke *R* ^2^ was quite low (Nagelkerke *R* ^2^=.052) and –2 log-likelihood was too high (–2 log-likelihood=1059.625). The analysis showed that the sociodemographic variables and health status alone did not satisfactorily predict whether persons were prone to use PRWs or not. An additional binary logistic regression (n=815) was calculated with the user and nonuser distinction as criterion and the variables gender, age, education, health status, feelings about the Internet, digital literacy, total daily Internet use in general, total daily Internet use for health-related information, importance of different sources for health-related information, appraisal of usefulness of PRWs, and trust of information on PRWs as predictors. The regression coefficients for gender and age were no longer significant. The regression coefficients were significant for the following variables: education (beta=.237, *P*=.005) implicating that a higher level of education predicted usage of PRWs, health status (beta=–.621, *P*=.001) demonstrating that a chronic disease predicted usage of PRWs, importance of family and pharmacist for health-related information demonstrating that lower importance of the 2 sources for health-related information had a significant impact on usage (family: beta=–.166, *P*=.02; pharmacist: beta=–.124, *P*=.046), trust in information on PRWs (beta=.329, *P*=.001), and the appraisal of usefulness of PRWs (beta=.216, *P*=.01) showing that higher trust and higher appraisal fostered usage of PRWs. Digital literacy (beta=.209, *P*=.05) and importance of the Internet for health-related information (beta=.141, *P*=.08) were also predictors, revealing that higher digital literacy and higher importance of the Internet were predictors for usage of PRWs. Nagelkerke *R* ^2^ was satisfying (Nagelkerke *R* ^2^=.248) with –2 log-likelihood=796.464.

**Table 4:** Binary logistic regressions for the user versus nonuser distinction.

Stepwise binary logistic regressions for different variables			Statistical fit	Standardized regression coefficients (beta)	*P*
**Step 1 (n=942)**					
	–2 Log-likelihood		1059.625		
	Pseudo *R* ^2^ (Nagelkerke)		0.052		
	**Predictors**				
		Constant (–1.561; *P*=.01)			
		Gender		.370	.02
		Age		–.012	.047
		Education		.271	<.001
		Health status		–.511	.001
**Step 2 (n=815)**					
	–2 Log-likelihood		796.464		
	Pseudo *R* ^2^ (Nagelkerke)		0.248		
	**Predictors**				
		Constant (–4.100; *P*<.001)			
		Gender		.293	.11
		Age		–.012	.11
		Education		.237	.005
		Health status		–.621	.001
		Feelings about the Internet and other Web-based applications in general		–.053	.60
		Digital literacy		.209	.05
		Total private daily Internet use		–.069	.12
		Total private daily Internet use for health-related information		.021	.70
		Importance of family for health-related information		–.166	.02
		Importance of friends for health-related information		.069	.32
		Importance of physician for health-related information		.071	.49
		Importance of pharmacist for health-related information		–.124	.046
		Importance of insurance agent for health-related information		–.024	.73
		Importance of Internet for health-related information		.141	.08
		Importance of books/journals for health-related information		.000	.99
		Usefulness of PRWs		.216	.01
		Trust of information on PRWs		.329	.001

## Discussion

### Principal Findings

One of the most significant findings to emerge from this study is that users and nonusers of PRWs differ according to several sociodemographic and psychographic variables and health status. Approximately one-quarter of respondents in our survey had used a PRW before. This is similar to the percentage described in the online study conducted by Emmert et al [20] in Germany in 2013 (25.3%) and it is higher than the percentage from a telephone survey of respondents in Germany in 2011 (10%) [41]. We agree with Emmert et al [20], who ascribed the difference between the 2 results to the survey method (telephone vs online panel). Our study revealed that younger people have more experience with PRWs. Further, more women than men, more highly educated people, and more persons with a chronic disease use PRWs. The findings of the current study are consistent with those of previous studies that suggested some sociodemographic groups are more likely to look online for health information than others [37]. The study by Emmert et al showed that women were more aware of German PRWs than men were. The same was true for people with higher health care utilization. Another finding of that study was that more women than men and more people with higher health care utilization had searched for physicians by using a German PRW [20]. Age was a significant (negative) predictor of PRW awareness in the study led by Galizzi et al in the United Kingdom [21].

However, the findings of the current paper go beyond those of the existing studies. The results of this investigation show that there are no significant differences between users and nonusers of PRWs in their daily Internet use, but users express more positive feelings toward the Internet and are more digitally literate than nonusers. Users and nonusers do not even differ in their daily Internet use for health-related information. The difference between users and nonusers may not lie in the quantity, but in the quality and content of Internet consumption because users see themselves as more digitally literate and have more positive feelings toward the Internet. It may be assumed that users search more efficiently for health-related information and use other sources and content of the Internet than nonusers of PRWs. This can be accentuated by the fact that the Internet is a more important source of health-related information for users than nonusers of PRWs. As expected, the usefulness of information and trustworthiness of PRWs is judged to be higher by users than nonusers. The 2 groups differ significantly in their future intentions concerning PRWs: Users are more prone to use a PRW in the future and to rate a physician online in the future.

This study has demonstrated that sociodemographic variables alone do not produce a satisfying model to predict usage or nonusage of PRWs. Instead, it is necessary to integrate additional psychographic variables and participants’ health status to predict usage or nonusage of PRWs to a more satisfying extent.

According to a causal perspective, higher education, a chronic disease, higher digital literacy, less importance on family and pharmacist for health-related information, higher importance on the Internet for health-related information, higher trust in information on PRWs, and a higher appraisal of the usefulness of PRWs are positive predictors of usage of PRWs. Other variables are not predictors of usage and nonusage (eg, gender and age), which is consistent with findings by Galizzi et al [21] who also found in their logistic regressions that gender and age had no effect on the intention to use PRWs. In our study, having a chronic disease lead to a higher probability of using PRWs. This is in-line with French and Italian studies demonstrating that respondents perceiving themselves as less healthy are more prone to use eHealth [20,42]. However, other studies (eg, Emmert et al [20]) did not find a significant impact of health status on awareness of searching on PRWs or the use of the Internet for health-related information [43,44]. Additional research is necessary to gain more insight into these divergent findings in the literature. In our study, a higher importance of family and pharmacist as an information source for health-related information predicted lower usage of PRWs. One explanation might be that there is a trade-off between the family and the pharmacist and PRWs as sources for health-related information. Respondents preferring the personal relationship of others as a source of health-related information are less prone to use PRWs. On the other hand, if someone ascribes high importance to the Internet (instead of personal relationships, for example) as a source of health-related information, he/she is also more prone to use PRWs. The latter is in-line with the results of Galizzi et al [21], who argued that the willingness to use PRWs is higher for individuals judging websites of hospital statistics as important sources of information.

The results suggest that the level of usage of PRWs is different in different population segments. It seems that current users of PRWs are younger, better educated, female, as well as individuals with a chronic disease. These segments may be innovators in the area of PRW usage, which could be a valuable insight for those interested in increasing PRW usage.

### Limitations

There are some limitations to this study. There is the possibility of selection bias among respondents, although random selection out of the database was held to minimize its likelihood and the recruitment rate was 64% for this online panel sample. Participants of an online sample may be more familiar with Internet-related topics [20], such as PRWs; therefore, it can be assumed that they have a higher awareness of PRWs than the average population. A demographic comparison of our sample showed that there were more respondents with a higher education than in the general population. An additional large randomized sample of the average population would certainly be desirable. But, as far as we know, no study has investigated psychographic differences in addition to the sociodemographic ones between users and nonusers of PRWs from the patients’ point of view. So, the results shed new light on the possibilities of boosting usage of existing PRWs or on the development of new PRWs.

### Practical Implications

Based on our results, communication concepts of PRWs should be tailored to the requirements of their users. The website design, the usability, and the accessibility of the PRWs and user-generated content should meet the users’ requirements for further usage of PRWs (eg, clear design of the PRWs, simple handling of the search functions when looking for a physician, introducing links to the websites of the physicians) [45,46]. Consideration should be given to using the innovators (female, better-educated, younger individuals that have experience with PRWs) as a personal communication source for others to spread the usage of PRWs. One might think about the kind of social media that are used by these user segments and tailor communication concepts for PRWs through social media according to the consumer habits of these segments. Reimann and Strech [12] argued that the use of PRWs will increase in the near future when the generation socialized with social media (eg, Facebook) reaches the age in which health questions and doctors become more important.

It should be kept in mind that all users who are satisfied with their experience with PRWs could function as promoters to diffuse their experience to new user segments, which can be seen as followers in this innovative area.

Results could also be interesting for physicians. Instead of rejecting PRWs, physicians should regard PRWs as an important source of recommendation. If physicians know about the sociodemographic and psychographic profiles of PRW users, they could invite specific patients belonging to these segments who are more likely to use PRWs, to rate them on PRWs, and to pass on their experiences to other patients on PRWs. Also, creators of PRWs should convince physicians of the advantages of PRWs, for example, that good ratings would enhance the possibility of winning new patients and broaden their patient base. Physicians could also use PRWs as a marketing instrument. In this vein, usage of PRWs could also be pushed from the physician side.

## Multimedia Appendix 1

Questionnaire PRWs.

## Abbreviations

GfK: Gesellschaft für Konsumforschung
GP: general practitioner
PRW: physician-rating website
