# Supplementary material for: Who Uses Physician-Rating Websites? Differences in Sociodemographic Variables, Psychographic Variables, and Health Status of Users and Nonusers of Physician-Rating Websites
Source: J Med Internet Res. 2014 Mar 31;16(3):e97. doi: 10.2196/jmir.3145 (PMC4004145; doi:10.2196/jmir.3145)
Supplement: Supplementary file 1 [file jmir_v16i3e97_app1.pdf]

ALLGEMEINE EINSTELLUNG GEGENÜBER DEM INTERNET / GENERAL ATTITUDE TOWARD THE INTERNET

1. **Welche Gefühle haben Sie gegenüber dem Internet und anderen webbasierten Anwendungen (z.B. Anwendungen am Smartphone oder Tablet-PC) im Allgemeinen?**

   What kind of feelings do you have toward the Internet and other web-based applications (e.g. apps on the smart phone or tablet) in general?

Very negative						Very positive	No answer
1	2	3	4	5	6	7	8

DIGITAL LITERACY

2. **Wie würden Sie sich auf einer Skala von 1 (Ich kenne mich überhaupt nicht aus) bis 7 (Ich kenne mich sehr gut aus), im Umgang mit dem Internet im Allgemeinen, selbst einstufen?**

   How would you rate your own Internet skills on a scale from 1 (I am not literate at all) to 7 (I am very literate)?

Not literate at all						Very literate	No answer
1	2	3	4	5	6	7	8

NUTZUNGSVERHALTEN

3. **Wenn Sie an Ihre Internetnutzung denken, wie viele Stunden nutzen Sie durchschnittlich das Internet für private Tätigkeiten?** (Bitte wählen Sie eine Antwortoption (pro Tag oder pro Woche oder pro Monat) aus, die Ihrer Nutzung am ehesten entspricht)
   Durchschnittliche Internetnutzung in **Stunden pro Tag** ___________
   Durchschnittliche Internetnutzung in **Stunden pro Woche** ___________
   Durchschnittliche Internetnutzung in **Stunden pro Monat** ___________

   If you think about your Internet usage, how many hours do you spend on the Internet on average for private purposes? (Please select one answer option (per day or per week or per month), which correspondsto your usage most closely)
   Average Internet usage **in hours per day** ___________
   Average Internet usage **in hours per week** ___________
   Average Internet usage **in hours per month** ___________

4. **Wie viele Stunden nutzen Sie durchschnittlich das Internet für die Suche nach gesundheitsorientierten Informationen?**
   (Bitte wählen Sie eine Antwortoption (pro Tag oder pro Woche oder pro Monat) aus, die Ihrer Nutzung am ehesten entspricht)
   Durchschnittliche Internetnutzung in **Stunden pro Tag** ___________
   Durchschnittliche Internetnutzung in **Stunden pro Woche** ___________
   Durchschnittliche Internetnutzung in **Stunden pro Monat** ___________

   How many hoursdo you spend on the Internet on average searching for health related information?
   Average Internet usage **in hours per day** ___________
   Average Internet usage **in hours per week** ___________
   Average Internet usage **in hours per month** ___________

5. **Welche Bedeutung haben für Sie die einzelnen Quellen, wenn Sie gesundheitsorientierte Informationen benötigen?**

		Überhaupt keine Bedeutung						Sehr hohe Bedeutung	Keine Antwort
F6_1	Familie	1	2	3	4	5	6	7	8
F6_2	Freunde	1	2	3	4	5	6	7	8
F6_3	Arzt/Ärztin	1	2	3	4	5	6	7	8
F6_4	Apotheker/in	1	2	3	4	5	6	7	8
F6_5	Versicherungsberater/in	1	2	3	4	5	6	7	8
F6_6	Internet	1	2	3	4	5	6	7	8
F6_7	Bücher/Medizinzeitschriften	1	2	3	4	5	6	7	8
F6_8	Andere Quellen	1	2	3	4	5	6	7	8

How important are the following sources for you, if you need health related information?

		Not important at all						Very important	No answer
F6_1	Family	1	2	3	4	5	6	7	8
F6_2	Friends	1	2	3	4	5	6	7	8
F6_3	Physician	1	2	3	4	5	6	7	8
F6_4	Pharmacist	1	2	3	4	5	6	7	8
F6_5	Insurance agent	1	2	3	4	5	6	7	8
F6_6	Internet	1	2	3	4	5	6	7	8
F6_7	Books/journals	1	2	3	4	5	6	7	8
F6_8	Other sources	1	2	3	4	5	6	7	8

REZENSIONEN / REVIEWS

6. Im Folgenden werden Weiterempfehlungsplattformen abgefragt. **Kennen Sie Plattformen im Internet, auf denen PatientInnen die Möglichkeit haben, ihren letzten Arztbesuch zu bewerten?** Ein Beispiel für eine solche Plattform wäre imedo.de.

   1 Ja   2 Nein   3 Keine Antwort

   Questions regarding rating websites will be asked below. **Do you know any websites, where patients have the opportunity to rate their physicians?**

   1 Yes   2 No   3 No answer

7. **Haben Sie schon einmal Informationen über einen Arzt / eine Ärztin auf einer solchen Plattform eingeholt?**

   1 Ja   2 Nein   3 Keine Antwort

   Have you ever gathered information on a physician from a PRW?

   1 Yes   2 No   3 No answer

8. **Haben Sie schon einmal einen Arzt / eine Ärztin selbst bewertet?**

1 Ja 2 Nein 3 Keine Antwort

Have you ever rated a physician on a PRW?

1 Yes 2 No 3 No answer

9. **Wenn nein, inwiefern können Sie sich vorstellen einen Arzt/eine Ärztin auf einer Empfehlungsplattform im Internet zu bewerten?**

Kann ich mir überhaupt nicht vorstellen						Kann ich mir sehr gut vorstellen	Keine Antwort
1	2	3	4	5	6	7	8

If „no“, can you imagine rating a physician on a PRW?

1 Yes 2 No 3 No answer

10. **Wie wahrscheinlich ist es, dass Sie in Zukunft eine solche Empfehlungsplattform für Ärzte/Ärztinnen nutzen werden?**

Überhaupt nicht wahrscheinlich						Sehr wahrscheinlich	Keine Antwort
1	2	3	4	5	6	7	8

How probable is it that you will use such a PRW in the future?

Not at all probable						Very probable	No answer
1	2	3	4	5	6	7	8

11. **Wie nützlich finden Sie solche Empfehlungsplattformen für die Arztsuche im Vergleich zu anderen Empfehlungsquellen (z.B. andere Ärzte, Familie, Bekannte etc.)?**

Überhaupt nicht nützlich						Sehr nützlich	Keine Antwort
1	2	3	4	5	6	7	8

How useful are PRWs in comparison to other recommendation sources (e.g. other physicians, family, friends etc.) from your point of view?

Not at all useful						Very useful	No answer
1	2	3	4	5	6	7	8

12. **Inwieweit vertrauen Sie den Informationen auf Empfehlungsplattformen für Ärzte und Ärztinnen?**

Ich habe überhaupt kein Vertrauen.						Ich habe sehr großes Vertrauen.	Keine Antwort
1	2	3	4	5	6	7	8

How much do you trust the information on PRWs?

No trust at all						Very high trust	No answer
1	2	3	4	5	6	7	8

DEMOGRAPHISCHE ANGABEN / SOCIODEMOGRAPHIC DATA

13. Haben Sie eine chronische Erkrankung?

1 Ja   2 Nein   3 Keine Antwort

Do you have a chronic disease?

1 Yes   2 No   3 No answer

D1 **Gender:**   [1] male
[2] female

D2_1 **Year of birth:** ______________

Höchste abgeschlossene Ausbildung:

1 Schüler in allgemeinbildender Schule (ohne Schulabschluss)
2 Haupt-/Volksschulabschluss ohne Lehre
3 Haupt-/Volksschulabschluss mit Lehre
4 Weiterführende Schule ohne Abitur (Realschulabschluss / Mittlere Reife)
5 Abitur / (Fach-) Hochschulreife ohne Studium
6 Abitur / (Fach-) Hochschulreife mit Studium
7 Promotion/ Habilitation
8 Keine Antwort

Highest education level:

1 Without school qualification
2 Secondary general school
3 Polytechnic secondary school
4 Intermediate secondary school
5 Matura examination
6 University degree
7 Postdoctoral degree / Professor
8 No answer
